# COVID-19 reinfections in Mexico City: implications for public health

**DOI:** 10.3389/fpubh.2023.1321283

**Published:** 2024-02-14

**Authors:** Guillermo de Anda-Jáuregui, Laura Gómez-Romero, Sofía Cañas, Abraham Campos-Romero, Jonathan Alcántar-Fernández, Alberto Cedro-Tanda

**Affiliations:** ^1^Instituto Nacional de Medicina Genómica, Mexico City, Mexico; ^2^Investigadoras e Investigadoras por México, Consejo Nacional de Humanidades, Ciencias y Tecnologías, Mexico City, Mexico; ^3^Centro de Ciencias de la Complejidad, Universidad Nacional Autónoma de México, Mexico City, Mexico; ^4^Escuela de Medicina y Ciencias de la Salud, Tecnológico de Monterrey, Mexico City, Mexico; ^5^Instituto Tecnológico de Estudios Superiores de Monterrey, Monterrey, Mexico; ^6^Innovation and Research Department, Culiacan, Mexico; ^7^Núcleo B de Innovación en Medicina de Precisión, Instituto Nacional de Medicina Genómica, Mexico City, Mexico

**Keywords:** SARS-CoV-2, COVID-19 reinfections, Mexico City, epidemiological surveillance, vaccination strategies

## Abstract

**Background:**

Since its appearance, COVID-19 has immensely impacted our society. Public health measures, from the initial lockdowns to vaccination campaigns, have mitigated the crisis. However, SARS-CoV-2’s persistence and evolving variants continue to pose global threats, increasing the risk of reinfections. Despite vaccination progress, understanding reinfections remains crucial for informed public health responses.

**Methods:**

We collected available data on clinical and genomic information for SARS-CoV-2 samples from patients treated in Mexico City from 2020 epidemiological week 10 to 2023 epidemiological week 06 encompassing the whole public health emergency’s period. To identify clinical data we utilized the SISVER (Respiratory Disease Epidemiological Surveillance System) database for SARS-CoV-2 patients who received medical attention in Mexico City. For genomic surveillance we analyzed genomic data previously uploaded to GISAID generated by Mexican institutions. We used these data sources to generate descriptors of case number, hospitalization, death and reinfection rates, and viral variant prevalence throughout the pandemic period.

**Findings:**

The fraction of reinfected individuals in the COVID-19 infected population steadily increased as the pandemic progressed in Mexico City. Most reinfections occurred during the fifth wave (40%). This wave was characterized by the coexistence of multiple variants exceeding 80% prevalence; whereas all other waves showed a unique characteristic dominant variant (prevalence >95%). Shifts in symptom patient care type and severity were observed, 2.53% transitioned from hospitalized to ambulatory care type during reinfection and 0.597% showed the opposite behavior; also 7.23% showed a reduction in severity of symptoms and 6.05% displayed an increase in severity. Unvaccinated individuals accounted for the highest percentage of reinfections (41.6%), followed by vaccinated individuals (31.9%). Most reinfections occurred after the fourth wave, dominated by the Omicron variant; and after the vaccination campaign was already underway.

**Interpretation:**

Our analysis suggests reduced infection severity in reinfections, evident through shifts in symptom severity and care patterns. Unvaccinated individuals accounted for most reinfections. While our study centers on Mexico City, its findings may hold implications for broader regions, contributing insights into reinfection dynamics.

## Introduction

In recent years, the world has faced unprecedented challenges posed by the COVID-19 pandemic. To combat the virus, extensive public health efforts, ranging from lockdowns and social distancing to the implementation of non-pharmaceutical interventions, have been crucial. Additionally, widespread vaccination campaigns have played a significant role in our collective response. These combined efforts have allowed us to move forward with a sense of normalcy from the most critical phase of the sanitary emergency.

However, despite international and national agencies announcing in early 2023 the ending of the public health emergency, the reality is that SARS-COV-2 is still in circulation and such announcements do not imply that COVID-19 is no longer a global threat. Crucially, populations where the virus propagated freely, and as viral variants continue to emerge, are progressively being more affected by SARS-CoV-2 reinfections.

Some studies have characterized the reinfection rate over different periods of time showing varying degrees of reinfection rates. In a retrospective study encompassing 238 US healthcare facilities between 1 June 2020 and 28 February 2021 a reinfection rate of 0.2% was observed. In another study, based on a cohort of 1,806 healthcare workers from a single, large, tertiary cancer center in India, the 52-week probability of reinfection was 2.2% with data cut-off on June 2021. By other hand, in a retrospective cohort analysis of the entire population of an Italian region including 1,293,941 subjects from the beginning of the pandemic to up to mid-February 2022 (follow-up period of 277 days) an overall reinfection rate of 6.1% was observed, after 18–22 months from the primary infection, the infection rate was still and 6.7% (1–3).

Reinfection risk has been shown to increase as the time to the first infection increases, reaching a maximum and stabilizing. Different time periods required to reach this stability have been reported from 277 days to 18 months at a maximum value of 6.7 and 18.86%, respectively (3, 4).

Understanding disease severity of reinfections compared to primary infection is important to anticipate the burden of public systems and to aid on decision-making. Several reports have found that previous infection gives protection against severe reinfections and that risk of hospitalization and deaths diminishes in reinfections (5, 6). A study found that the risk of having a severe reinfection was extremely low in persons previously infected compared to uninfected persons (1%) (5). Importantly, protection achieved by primary infection is comparable with that offered by vaccines (7).

However, the interpretation and application of all findings are complicated since virus variants have diverged over time and geographic location, vaccine distribution per vaccine type has been uneven around the globe, local vaccine administration strategies have sometimes been focused on specific population sectors, and vaccine efficiency per type is different. Besides, reports do not always categorize reinfection rate per vaccination status (8–10).

In the case of Mexico, all public institutions and authorized laboratories were required to register confirmed SARS-CoV-2 cases in a federal database named SISVER along with patient demographic information, date of onset of symptoms, symptomatology, vaccination-related information and other relevant clinical data. While this is one of the largest available COVID-19 case datasets, it’s important to note data limitations. Testing practices evolved over time, starting with PCR-based testing for severe cases and later expanding to include asymptomatic individuals. Mexico City conducted robust testing; however, it should be noted that there were no random sampling efforts, and all cases were detected due to the patient seeking medical attention or diagnosis. As such, data capture issues, including reporting delays and under-reporting, are acknowledged challenges. Despite these limitations, it’s important to emphasize that employing appropriate statistical methods can yield valuable insights.

As the pandemic continues to evolve, reinfection cases are becoming increasingly common due to the majority of new infections falling into the reinfection category. In this study, we conducted a comprehensive analysis of the prevalence of SARS-CoV-2 variants over time in Mexico City, spanning from the onset of the pandemic to the conclusion of the COVID-19 health emergency in Mexico. Additionally, we delved into the specifics of reinfection cases within Mexico City, examining factors such as the time intervals between infections. Furthermore, we explored the correlation between the reinfection rate and shifts in the predominant SARS-CoV-2 variant. Lastly, we conducted an in-depth examination of clinical outcomes, infection severity, and vaccination status in individuals experiencing reinfections.

## Methods

### Data collection

This is a retrospective study analyzing data from the SISVER (Respiratory disease epidemiological surveillance system) database, the Mexican federal government central COVID-19 case reporting system. Briefly, this is a “line-list” case dataset, where each row corresponds to a single case. Only official public reporting institutions (either at the state or federal level) report to this system. As a member of the Federal Health System, our institution has access to the full database, including 130 variables for each case. For this work, we considered the following inclusion criteria: (i) confirmed COVID-19 cases, having a positive result through either PCR or antigen testing, (ii) records collected in medical units or testing sites located within the boundaries of Mexico City; and only considering cases with a reported residence in Mexico City and (iii) cases collected up to 2023-05-13 (end of epidemiological week 2023–19).

We used as a secondary data source data collected by Salud Digna A.C. laboratories (SD). SD is a well-established healthcare provider, their data collection and reporting processes are likely to be consistent and reliable which is crucial for ensuring the quality and accuracy of the data used in the study. SD has laboratories in Mexico City covering all territories inside the city. SD data included (i) confirmed cases of COVID-19 by PCR per week, (ii) total number of cases analyzed and (iii) number of reinfections per week. SD data included cases collected up to 2022-07-16 (end of epidemiological week 2022–28). The inclusion of data from SD did not preclude the possibility of using other data sources from healthcare providers, however the choice was based on the availability, quality and coverage of data that SD could offer in the context of our specific research on COVID-19 reinfection dynamics in Mexico City.

This study was approved by the ethics and research committees of the Instituto Nacional de Medicina Genómica (CEI/1479/20 and CEI 2020/21).

### Identification of reinfections in the dataset

We define as a reinfection any secondary case with symptoms onset date at least 90 days later than the primary infection symptom onset date, based on the cutoff date used in (11–14). Symptom onset date was used as the main criteria to diminish the noise caused by reporting delays. Tertiary and quaternary infections were also identified if they had symptoms onset date at least 90 days later than the previous infection. We used the Unique Key for Population Registry (CURP), which is a unique population identifier recorded in the dataset to uniquely identify each individual.

### Wave definition

The COVID-19 epidemic in Mexico, as in other parts of the world, has exhibited periods of high transmission or “waves.” The definition of such waves has been difficult due to changes in detection criteria. Intuitively, a wave can be thought of as a period that starts with an increase in the number of cases, and ends when that number of cases drops back to a baseline. To quantitatively assess this, we use the focus on the rate of change of hospitalized cases, since this indicator is less biased by sampling issues. Therefore, we used a methodology first proposed in (15). Briefly, the authors looked at the (7-day rolling average) daily hospitalization counts, and considered a wave as beginning on the epidemiological week when the second derivative of this time series became positive, and ending on the epidemiological week when the second derivative of the time series became negative. We defined the periods between these waves as “interwave periods.” The start weeks for each wave and interwave periods are shown in Table 1.

**Table 1:** Start weeks for each wave and interwave periods.

Start date (Epidemiological week)	Period
2020–10	Wave 1
2020–33	Interwave period 1
2020–44	Wave 2
2021–13	Interwave period 2
2021–24	Wave 3
2021–42	Interwave period 3
2021–50	Wave 4
2022–15	Interwave period 4
2022–21	Wave 5
2022–36	Interwave period 5
2022–46	Wave 6
2023–06	Interwave period 6

### Variant prevalence analysis

#### Sample collection

Nasopharyngeal swab were collected by a trained clinician with a flexible nylon swab that was inserted into the patient’s nostrils to reach the posterior nasopharynx. The swab was left in place for several seconds and slowly removed while rotating. The swab was then placed in 2 mL of sterile viral transport medium. Swabs from both nostrils were deposited in a single viral transport tube, taken to a clinical laboratory, and processed immediately.

Total nucleic acid was extracted from 300 μL of viral transport medium from the NPSs or 300 μL of whole saliva using the MagMAX Viral/Pathogen Nucleic Acid Isolation Kit (Thermo Fisher Scientific, Waltham, MA, United States) and eluted into 50 μL of elution buffer.

For SARS-CoV-2 RNA detection, 5 μL of RNA template was tested using TaqPath master mix (Thermo Fisher Scientific, Waltham, MA, USA). All tests were run on a Thermo Fisher ABI QuantStudio 5 real-time thermal cycler (Thermo Fisher Scientific, Waltham, MA, United States). Samples were selected for inclusion in this study based on viral Ct < 30.

#### Illumina sequencing

The libraries were prepared using the Illumina COVID-seq kit, following the manufacturer’s instructions. First-strand synthesis was carried out with RNA samples, the synthesized cDNA was amplified using ARTIC primers v4, then was tagmented and adapted using IDT for the Illumina Nextera UD Indices Set A, B, C, D (384 indices) (Illumina, San Diego, CA, United States). Dual-indexed pair-end sequencing with a 150 bp read length was carried out on the NextSeq 2000 platform (Illumina, San Diego, CA, United States).

#### Oxford nanopore sequencing

Libraries were prepared according to ARTIC Midnight protocol PCR tiling of SARS-CoV-2 virus with rapid barcoding kit (SQK-RBK110.96) and sequenced on the GridION sequencing platform. We use the PCRT_9125_v110_revE_24Mar2021 protocol. 800 ng of DNA library was loaded into a primed R.9 flow cell (FLO-MIN106). MinKNOW software v.21.11.7 (Oxford Nanopore Technologies, Oxford, Oxfordshire, United Kingdom) was used to collect raw sequencing data and basecalling. Oxford-Nanopore Raw Data Processing and Sequencing Data Quality Assessment Basecalling and barcode demultiplexing were performed following the ARTIC protocol.^1^ We regularly upload genomic and metadata information to GISAID (16). The findings of this study are based on metadata available on GISAID.^2^

During the analysis period, a total of 23,722 genomes from Mexico City were uploaded to GISAID, out of which 16,160 were sequenced at INMEGEN, i.e., 70% of the SARS-CoV-2 genomes reported to GISAID were sequenced and uploaded by INMEGEN.

### Clinical severity characterization

To assess whether post-primary infections are milder than the original infection, we used the symptom information contained in the SISVER database. The dataset has 20 binary variables to encode symptoms presented by the patient at the time of admission. Based on previous work (17) we labeled cases as severe if they reported any of the following symptoms: cyanosis, dyspnea, polypnea, or sudden symptom onset; or if the patient required intubation. Cases that presented no symptoms were labeled as asymptomatic. The rest of the cases were labeled as mild infections.

## Results

### Each wave of SARS-CoV-2 infections was characterized by a different viral variant

**Figure 1:**
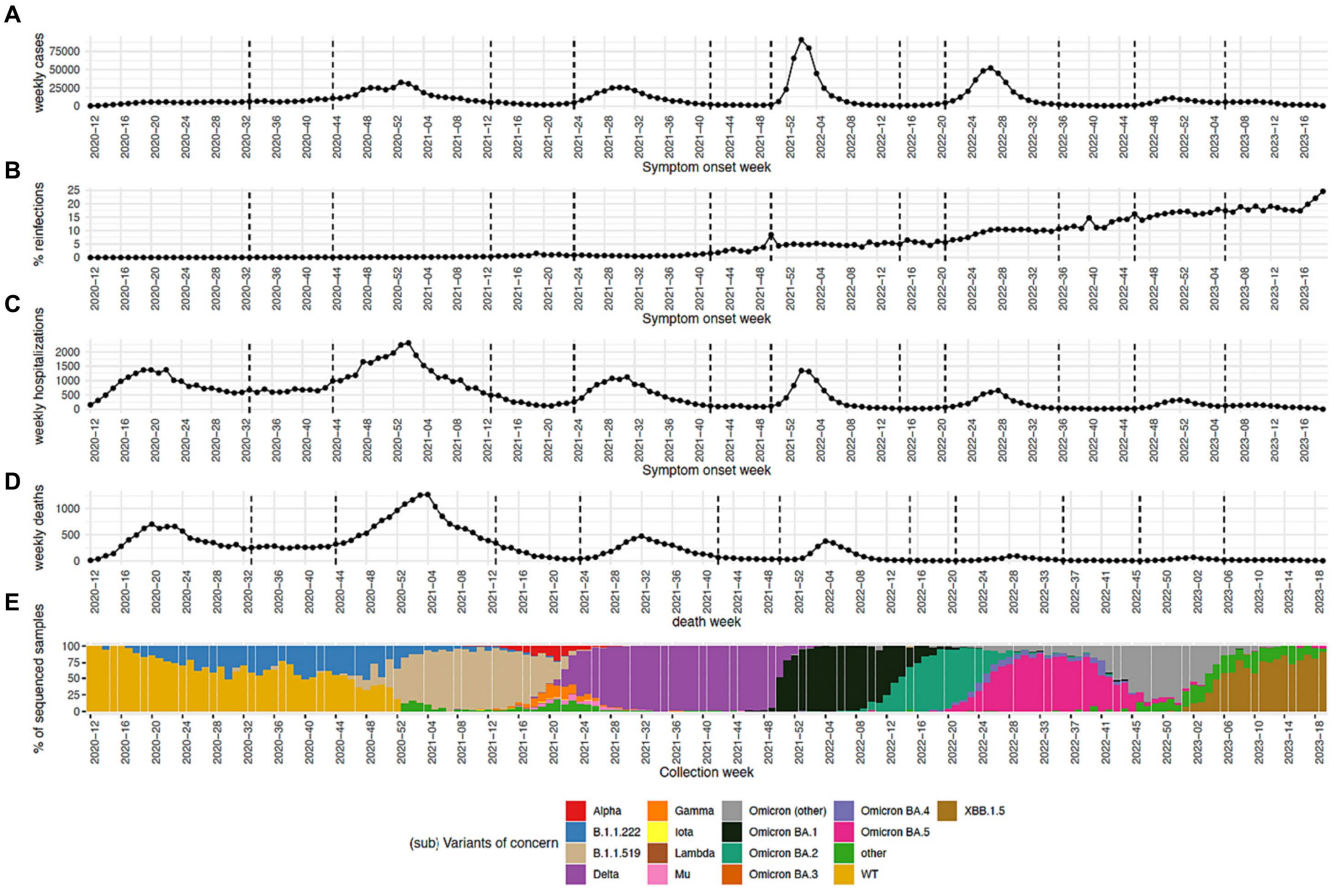
**(A)** Number of cases per epidemiological week. **(B)** Percentage of cases that belong to reinfections. **(C,D)** Percentage of cases that turned out in either death or hospitalization, respectively, are shown. **(E)** Percentage of sequenced samples per SARS-CoV-2 variant per epidemiological week. The group Omicron (other) includes any other Omicron sub-variants reported in this time period.

In our study, we conducted an analysis of the prevalence of SARS-CoV-2 variants over time in Mexico City, with a focus on their impact on primary infection and secondary (reinfection) epidemic curves. The predominant SARS-CoV-2 variant exhibited dynamic fluctuations across different waves of the pandemic. Moreover, the duration and intensity of each wave, as reflected by the number of cases, hospitalizations, and deaths, showed significant variations (Figure 1; Supplementary Figure S1; Supplementary Data Sheet 2).

During wave 1 (epidemiological week 2020–10 to 2020–32) the WT SARS-CoV-2 lineage started as the predominant lineage with a prevalence of 100%, this lineage was rapidly displaced by B.1.1.222 which became the predominant SARS-CoV-2 variant with a maximum incidence of 51.9%. B.1.1.222 was displaced by B.1.1.519 reaching a maximum incidence of 95.58% during wave 2 (epidemiological week 2020–44 to 2021–12) followed by the introduction of Delta which was the most prevalent in wave 3 reaching a maximum incidence of 100% (epidemiological week 2021–24 to 2021–41). Delta continues to show a prevalence of 100% during some time of interwave 3. After that, Omicron BA.1 reached a 99.7% prevalence during wave 4 (epidemiological week 2021–50 to 2022–14). Omicron BA.1 was displaced by Omicron BA.2 which reached a prevalence of 93.2% during the interwave 4 period. Omicron BA.2 continued to be the most prevalent variant at the start of wave 5 (epidemiological week 2022–21 to 2022–35) reaching a maximum prevalence of 80.9% followed by the rapid introduction and spread of Omicron BA.5 which reached a prevalence of 88.63% during this wave. During the interwave period 5 and wave 6 omicron subvariants were being in constant rechange. As such, during this period a dominant Omicron subvariant was not observed, but rather an ensemble. We called this ensemble as Omicron (“other”). Omicron BA.5 was replaced by the ensemble Omicron (“other”) during the interwave period, this showed a maximum prevalence of 87.5% during wave 6 (epidemiological week 2022–46 to 2023–05) and was displaced by the XBB.1.5 variant which reached a maximum prevalence of 91.7% during the interwave 6 period.

**Table:** 

2021–50	Wave 4
2022–15	Interwave period 4
2022–21	Wave 5
2022–36	Interwave period 5
2022–46	Wave 6
2023–06	Interwave period 6

Number of cases reached a maximum during wave 4 corresponding to 91,040 cases in week 2022–02 for SISVER data and 38,849 for SD data, however the number of weekly hospitalizations and deaths for SISVER data reached their maximum value close to the start of the pandemic during wave 2 with a maximum value of 3,323 and 1,270, respectively. The number of weekly cases, hospitalizations and deaths have displayed a decreasing behavior if the maximum number of cases at wave 4, 5 and 6 is considered, as is shown in Figure 1.

In this study, the percentage of reinfection was calculated as the number of infections that were considered as reinfections over the total number of infections in a given epidemiological week. We observed that the percentage of reinfection has been steadily increasing since 2021 epidemiological week 43, reaching a maximum of 24.6% by 2023 epidemiological week 19. This week corresponds to the end of the sanitary emergency in Mexico and consequently to the last week analyzed in this study.

### Reinfections occurred mostly in the Delta and Omicron waves.

We analyzed the time between infections for all patients having more than one confirmed infection in the SISVR database (consecutive confirmed cases). The distribution of the time between infections can be seen in Supplementary Figure S2. This distribution shows a cyclic pattern displaying periods of time between infections with a high number of infections followed by periods of time between infections with a low number of infections, these cyclic peaks of periods of time between infections which could be related to the cyclic pattern of infection rate throughout the pandemic characterized by periods of high intensity followed by low intensity periods. We noticed a large number of persistent infections recorded in the database, i.e., confirmed cases with a time of separation of few days (minimum 0 days), we observed the largest peak of persistent infections to be between 0 and 5 days since index infection which almost certainly belong to the same infection registered by different institutions or different departments from the same institution. This noisy behavior could be caused by the difficulty of controlling a national resource during the sanitary emergency.

Based on previous studies, we defined a reinfection as a non-primary infection occurring on the same patient with at least 90 days of separation (11, 13, 18). We considered the date of onset of symptoms as the day of infection. Our infection dataset included 147,189 infections throughout the pandemic occurring on 72,400 patients. Each one of these patients experienced a primary infection followed by a total of 74,789 non-primary infections (reinfections). Our dataset included 70,074 patients which experienced one and only one reinfection. For all analysis we only these patients as subsequent reinfections could have a distinctive behavior. The general breakdown of our infection dataset stratified by sex and age group is shown in Table 2 along with the number of infections, the number of primary infections and the number of non-primary infections.

**Table 2:** Breakdown of the reinfection dataset.

	Male	Female	Total
Number of infections			
By age group	56,632 (50.7)	90,557(50.88)	147,189 (50.81)
0–9	864 (46.76)	748 (46.79)	1,612 (46.77)
10–19	2,661 (46.22)	3,118 (44.54)	5,779 (45.28)
20–29	12,562 (47.92)	17,790 (48.38)	30,352 (48.19)
30–39	15,153 (51.54)	23,497 (50.87)	38,650 (51.14)
40–49	12,274 (50.98)	22,308 (51.12)	34,582 (51.07)
50–59	8,278 (52.39)	15,900 (53.06)	24,178 (52.83)
60–69	3,505 (55.17)	5,605 (54.66)	9,110 (54.86)
70+	1,335 (53.71)	1,591 (54.93)	2,926 (54.37)

We analyzed the separation time between the primary infection and the reinfection. This number was smoothed by assigning each infection to a wave or interwave period and calculating the separation time between the start of the two assigned periods. We obtained an average separation time of 59.6 weeks. We found no statistically significant linear correlation between the percentage of reinfections occurring between each combination of periods to the time of separation between periods (Supplementary Figure S3, R-squared = 0.01, *p* = 0.280).

**Figure 2:**
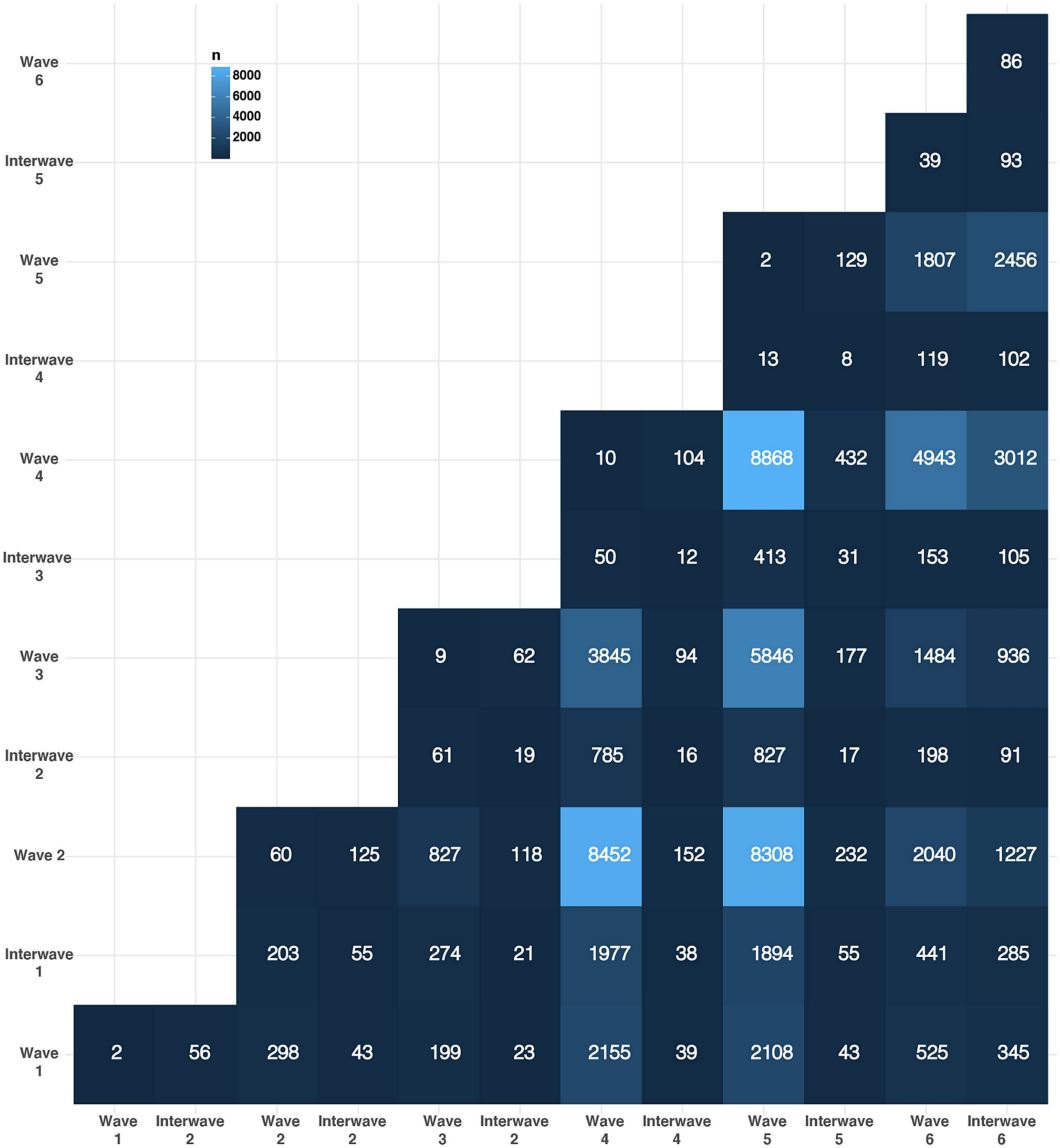
The number of reinfections across epidemic waves is shown. The y axis represent the time period of the index infection, the x axis represents the time period of the reinfection and the color of the tile indicates the number of reinfections in that specific combination of time periods.

We calculated the percentage of reinfections occurring between all waves and interwave periods of the pandemic. The largest fraction of reinfection corresponded to 8,868 (12.7%) patients with index infections at wave 4 that were reinfected at wave 5 corresponding to 23 weeks of separation between infections. Followed shortly by 8,452 (12.1%) and 8,308 (11.9%) patients with index infections at wave 2 who were reinfected at wave 4 and patients with index infections at wave 2 who were reinfected at wave 5 corresponding to 59 and 82 weeks of separation between infections, respectively (Figure 2; Supplementary Figure S4).

Reinfections usually involve wave 4 and wave 5 of the pandemic with 49.4 and 46.6 percentage of reinfections whose either index infection or reinfection occurred during these periods even when only 12.7% of reinfections occurred specifically between these two periods. Interestingly, wave 4 was the one with the highest number of observed cases and wave 5 has been the only wave with more than one SARS-CoV-2 variant with a prevalence higher than 80% at different epidemiological weeks (Omicron BA.2 followed by Omicron BA.5).

### Clinical outcome and severity of infection diminishes during reinfections

We analyzed the clinical outcome, e.g., indicated by the type of patient care: ambulatory vs. hospitalized, which is indicative of infection severity associated with reinfection. In Table 3 we present the number of reinfections per each clinical outcome, e.g., those that did not change clinical status during reinfection and those that transitioned from ambulatory to hospitalized and from hospitalized to ambulatory clinical outcome during reinfection. We found that in most cases 96.7% stayed as ambulatory in both infections. 2.53% patients were considered ambulatory during the reinfection even when they were hospitalized during the index infection. Only 0.597% presented and increased severity during its reinfection. Finally, 0.198% were hospitalized in both infections. This data suggests a diminished severity of infection during reinfection. However, this phenomenon could be related with vaccination since most not primary infections occurred during and after wave 4 and vaccination started in wave 2 and has continued over time.

**Table 3:** Transitions of type of patient care during reinfection.

Index infection	Reinfection	*n*	%
Ambulatory	Ambulatory	67,745	96.7
Hospitalized	Ambulatory	1772	2.53
Ambulatory	Hospitalized	418	0.597
Hospitalized	Hospitalized	139	0.198

We also analyzed the severity of symptoms classified as asymptomatic, mild or severe during the index infection and reinfection (Table 4). Most SARS-CoV-2 patients present a mild infection regardless of the type of infection: 90.6 and 93.2% mild infections in the case of index infections and reinfections, respectively. Asymptomatic infections are not common in the federal SISVER database accounting for 6.67 and 6.05% for index infection and reinfection, respectively. However, this could be an underestimate given that most data in the database is derived from hospital and clinical centers that receive symptomatic patients. So, this number may not properly reflect the rate of asymptomatic infections in the general population. 87.73% reinfections did not change severity of symptoms during reinfection: 1.15, 85.4, and 0.19% stayed as asymptomatic, mild or severe, respectively; 6.05% showed an increase in severity, i.e., transitioned from asymptomatic to mild or severe or from mild to severe; and 7.23% showed a reduction in severity, i.e., transitioned from severe to mild or asymptomatic or from mild to asymptomatic. Besides, in most cases reinfection tends to show a reduction in severity regardless of the wave in which the index infection or reinfection occurred (Supplementary Figure S5). This data is consistent with the diminished severity of infection during reinfection observed when the type of patient care is considered.

**Table 4:** Transitions of disease severity during reinfection.

Disease severity index infection	Disease severity reinfection	*n*	%
Asymptomatic	Asymptomatic	805	1.15
Asymptomatic	Mild	3,834	5.47
Asymptomatic	Severe	35	0.05
Mild	Asymptomatic	3,313	4.73
Mild	Mild	59,829	85.4
Mild	Severe	372	0.531
Severe	Asymptomatic	122	0.174
Severe	Mild	1,634	2.33
Severe	Severe	130	0.186

### Vaccination status for reinfection

We decided to explore whether reinfections occurred in vaccinated or unvaccinated individuals. The Mexican vaccination strategy has several possible confounding factors: it used a large array of vaccine products and individuals could not choose the type of vaccine that they would get, but were assigned one based on their age group and place of residence. Also, different vaccine brands were used over different time periods which could cause an unintended grouping by SARS-CoV-2 variant. So, we decided not to stratify the data per vaccine brand or by any other factor to avoid any unintended grouping by age group, place of residence or SARS-CoV-2 variant. Also, complete vaccination data over the general population indicating which population groups were vaccinated with specific vaccine brands is not publicly available.

We labeled cases as unvaccinated, if they had not received a vaccine previous to their infection; partially vaccinated, if they had received only one dose of a two-dose vaccine; vaccinated, if the infection occurred after receiving two doses; or boosted, if the patient had received additional vaccine doses. For this analysis we included all patients with one and only one reinfection that counted with information about their vaccination status at the time of the index infection and at the time of reinfection (*n* = 61,660 patients).

**Figure 3:**
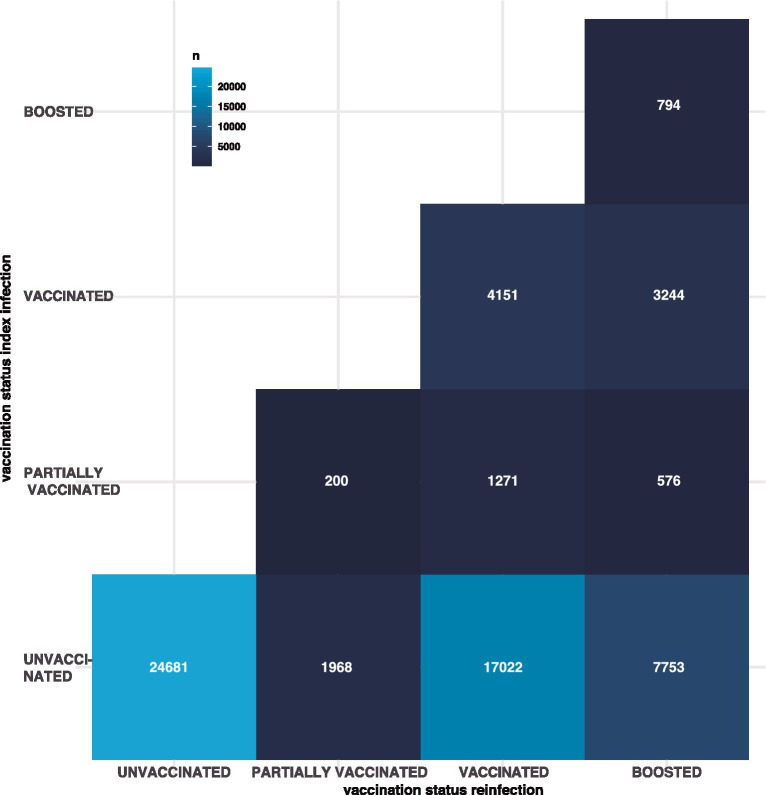
The number of reinfections per each combination of vaccination status. The y axis represent the vaccination status of the index infection, the x axis represents the vaccination status of the reinfection and the color of the tile is proportional to the number of reinfections for that specific combination. Vaccination satus: unvaccinated, if they had not received a vaccine previous to their infection; partially vaccinated, if they had received only one dose of a two-dose vaccine; vaccinated, if the infection occurred after receiving two doses; or boosted, if the patient had received additional vaccine doses.

Most index infections occurred at unvaccinated patients (51,424 out of 61,660) which is expected given that most index infections occurred at wave 2 before the national vaccination efforts reached a significant fraction of the population. From these infections, the highest number of reinfections have occurred in unvaccinated patients (48%), followed shortly by fully vaccinated patients (33.1%). Regardless of the patient vaccination status we observed the same trend, with reinfections being more common in unvaccinated patients (40%), followed shortly by vaccinated patients (36.4%) (Figure 3). Noteworthy, most reinfections occurred after wave 4 when the national vaccination efforts had reached 62.7% of the general population (19). Interestingly, 20.1% percent of reinfections occurred in patients with a vaccine boost dose. Noteworthy, the only vaccination data available for this study was the metadata recorded at the SISVR database which only encompass confirmed SARS-CoV-2 cases, in the case of Mexico vaccination coverage for the general population is not publicly available. Nonetheless, a comparison of vaccination status in reinfected patients through time against the coverage of the national vaccination campaign would be extremely useful to investigate the effect of vaccination over reinfection.

### Vaccination delays time between infections

Vaccination serves as a fundamental tool in the fight against COVID-19, underpinning public health policies worldwide. Originally designed to reduce severe outcomes, vaccines have also shown promise in mitigating symptomatic infections. In light of this, our study aims to assess how vaccination status influences reinfection patterns, offering valuable insights for pandemic management. In this work, we focused particularly on two scenarios: the effect of vaccination in people who were unvaccinated during their first infection, and the effect of boosters on people who were infected after a first vaccination (so called breakthrough infections.

#### Vaccination after an unvaccinated first infection delays the time between infections

**Figure 4:**
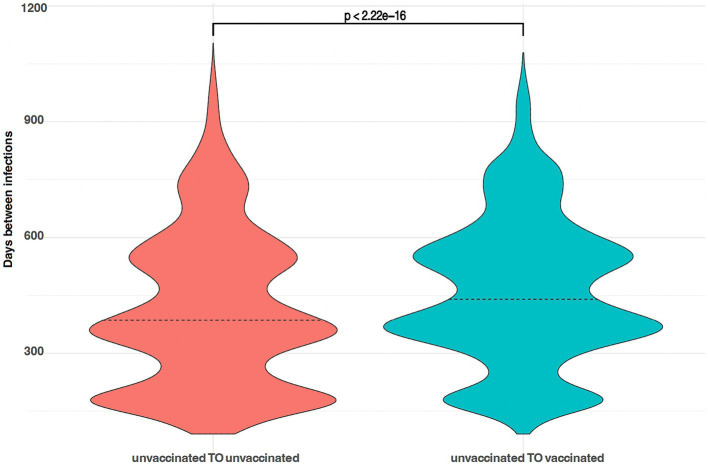
Violin plots showing the (multimodal) probability density function of time between infections; left, people unvaccinated before and after their first infection; right, people unvaccinated before their first infection and vaccinated before their second infection.

The temporal patterns of reinfections reveal a multimodal distribution, reflective of a dynamic process wherein the likelihood of infection, and consequently, reinfection, fluctuates with changing transmission rates. However, notable disparities emerge when comparing the median intervals between infections across populations. Specifically, the unvaccinated cohort exhibits a median time of 413 days between infections, contrasting with 460 days for the population who received vaccination following their initial infection. This divergence proves statistically significant, as confirmed by the Wilcoxon rank test (Figure 4).

#### Repeated vaccination after infection may increase protection against symptomatic infection

**Figure 5:**
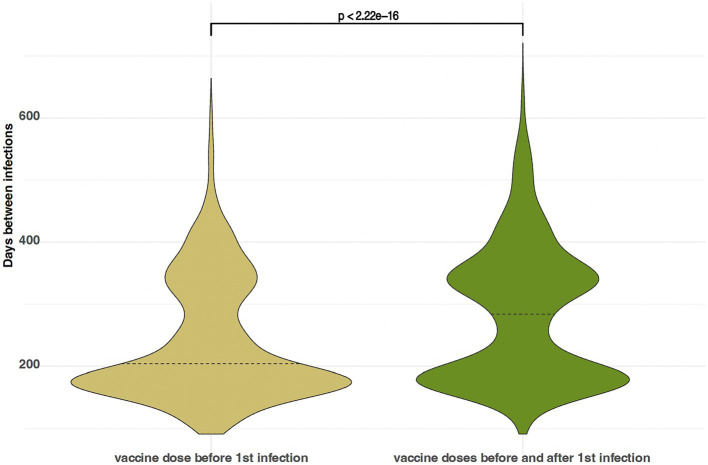
Violin plots showing the (multimodal) probability density function of time between infections; left, people vaccinated before their first infection, and not boosted afterwards; right, people vaccinated before first infection and boosted before their second infection.

In our assessment of populations who received vaccination before their initial infection, and later either received or did not receive a booster dose, we observe a clear bimodal pattern in the time between reinfections. This outcome is as expected, given that vaccines were introduced after the second wave of the pandemic. Additionally, we find a noteworthy difference in the median durations between infections in these groups. For those who received a booster vaccine dose after their initial infection, the median time between reinfections is 285 days, while the unboosted population shows a median interval of 240 days. Importantly, these differences are statistically significant, as confirmed by the Wilcoxon rank test (Figure 5).

These analyses confirm that vaccination provides protection against symptomatic reinfections, extending the time between infections. Boosters also play a vital role in delaying reinfections, even for those with previous breakthrough cases. Our findings align with recent studies on hybrid immunity (20) and emphasize the importance of regular booster shots for effective virus control; as they have a better effect of delaying symptomatic infection than a primary series alone. However, given the complexity of infection risk, determining an optimal vaccination schedule based solely on these findings is challenging. Further research is needed to refine such strategies.

## Discussion

For some viruses, the first infection generates lifelong immunity preventing reinfections, for seasonal coronaviruses this immunity is shorter, generating reinfections within the first 6 to 12 months (21). The ability of SARS-CoV-2 to mutate and generate new variants has led to reinfections worldwide. The first case of reinfection occurred in August 2020 (22), and the number of cases will continue to increase as new variants are generated that evade the immune response previously generated by another variant. This paper is the first, and largest, to cover cases of COVID-19 reinfection in Mexico City, offering insight toward vaccination dynamics in the context of SARS-CoV-2 reinfections.

In Mexico City, we identified six waves of infection each one displaying a different most prevalent SARS-CoV-2 variant. The first and second waves were caused by B.1.1.222 and B.1.1.519, respectively; variants that only circulated with high prevalence in Mexico (17) and United States, while in other parts of the world there was a diversification of variants during the same period of time. The SARS-CoV-2 variants with the highest prevalence during the waves 3, 4, 5 and 6, i.e., Delta, Omicron BA.1, Omicron BA.2 and Omicron BA.5, respectively, also caused waves of infections around the world (23).

The reinfection rate was 4.54% (74,789 reinfections of 1,648,061 positive cases) for the whole analysis period. In comparison with other studies (0.7–5.9%), this reinfection rate is similar to the average (3%) (11–14, 24–26).

It should be noted, however, that this rate increased as the pandemic continued; nearing 25% of all weekly cases by the end of the analysis period. Of the 70,074 reinfections analyzed, 65% (*n* = 45,553) occurred in Omicron BA.1 and BA.5 waves of infection, its variants already recognized for the gain of amino acid substitutions in spike protein (L452Q, L452R and F486V) associated with evasion of immune response and high affinity to ACE2 receptor (27, 28). The average number of days between primary and secondary infection in patients in Mexico City was 59.6 weeks, the period reported in a meta-analysis of 577 reinfection cases in 22 countries was 63 weeks (29).

Reinfections represent almost a quarter of newly diagnosed cases and they are becoming increasingly frequent, therefore becoming a bigger component of the public health impact of COVID-19 in Mexico City. There is an inherent complexity to the mechanisms behind reinfection, as there is an interplay between factors such as mitigation measures, non-pharmacological and pharmacological (vaccine) interventions, and the adaptation of the infectious agent itself (30). Our findings show that, non-vaccination, but also longer times since last vaccination, are associated with greater risk of reinfections. Given the generation of new variants that evade the immune response previously generated by vaccination or infection such as BA.2.75, BQ.1, BQ.1.1 and XBB, variants newly identified in November 2022 and already circulating worldwide with positive *in vitro* assays for evasion of antibody-mediated immune response (31–33).

Another important factor to consider when studying reinfections would be vaccination platform or brand. In this study, we did not assess the effect of vaccination type over reinfection due to lack of this information in our source data. However, we recognize the importance of these factors in the overall understanding of the dynamics of COVID-19. Almadhi et al. offers a detailed perspective on the efficacy of different COVID-19 vaccines over reinfection rate (34). In this study, they analyzed reinfections data taking into consideration the specific type of vaccine received, providing valuable insight into how different vaccines may influence the likelihood and severity of reinfections. These findings underline the need for future research that integrates, with more detail, vaccination data in the analysis of COVID-19 reinfections.

Only 0.597% of outpatients required hospitalization for their second infection; we have no further information about the diagnosis of hospitalized patients and/or the symptoms they presented. In a study of reinfections it was observed that 63 patients in their second infection presented: pneumonia and acute renal damage (14), while in another study 48 patients debuted with respiratory failure, thromboembolism and sepsis (35).

In relation to antibody immunity, our study shows that reinfections were more frequent in unvaccinated individuals and highlights changes in the severity of symptoms between the first infection and reinfection. This underscores the importance of a robust immune response to protect against reinfection, particularly in a context where vaccines were not yet available as mentioned in a 2022 study. Although it does not focus directly on virus variants, the study implicitly suggests that variants capable of evading the immune response may increase the risk of reinfection, especially if the initial immune response is weak (36).

SARS-CoV-2 reinfection dynamics have been described by multiple research groups throughout different countries. In relation to a study conducted in Bulgaria, notable differences are observed compared to our research in Mexico City. While the Bulgarian study highlighted a moderate reduction in severe outcomes during reinfections, emphasizing the protective role of vaccination, our analysis in Mexico revealed a continuous increase in reinfection rates, particularly during later waves dominated by Omicron variants. Furthermore, in our study, vaccination status was a significant factor, with a higher proportion of reinfections in unvaccinated individuals. This difference underscores the varied impact of vaccination strategies and specific epidemiological characteristics in different regions (37).

On the other hand, when comparing our study with research conducted in Ireland and the United States, we find that while the Irish systematic review and the US study provided a broader view of reinfection risk and the duration of post-infection immunity, our study in Mexico City offered a more detailed and localized perspective. Our analysis placed special emphasis on the dynamics of reinfections in relation to different pandemic waves and the prevalence of various virus variants, as well as the impact of vaccination on these dynamics. These differences highlight the importance of considering specific geographical and demographic contexts when evaluating COVID-19 reinfection patterns (38, 39).

It is known that the accumulation of SARS-CoV-2 infections results in a hazard ratio of hospitalization and death of 3.32 and 2.17, respectively (40). Accumulation of sequelae in multiple organs (lung, cardiovascular system, gastrointestinal system, kidneys, musculoskeletal and neurological), as well as mental health damage have been reported (41). Given that SARS-CoV-2 will continue to mutate for years to come, new variants with the potential to generate reinfections will continue to emerge, which could lead to a greater burden on the public health system given the comorbidities that occur in patients with reinfection, and its prevention will be one of the greatest future challenges in public health. In the absence of truly sterilizing vaccines (preferring vaccines designed for the most recent viral variants) that block transmission with a lasting effect, continued transmission may happen (42, 43) improving the burden of reinfections. As such, multilayered risk reduction approaches combining non-pharmaceutical (masking, ventilation, social distancing in periods of higher transmission) and the evaluation of recurrent vaccination may still be needed in the foreseeable future (44), as the effect of previous infection alone may not be sufficient to drive the pandemic away.

This study on the dynamics of COVID-19 reinfections in Mexico City provides crucial insights for Mexico, highlighting the need for public health strategies that complement vaccination, such as genomic surveillance. The findings inform policy decisions and vaccination strategies adapted to the evolution of virus variants and vaccine efficacy. The research contributes to the understanding of how viral variants influence reinfections, essential for health resource planning and to anticipate hospital care needs. Furthermore, it emphasizes the importance of continuously reevaluating vaccination strategies and suggests areas for future research, such as the long-term impact of reinfections and the efficacy of different vaccination regimes, valuable both for Mexico and for the global understanding of the pandemic.

## Data availability statement

The original contributions presented in the study are included in the article/Supplementary material. GISAID accessions numbers can be found in Supplementary Data Sheet 3.

## Author contributions

GA-J: Conceptualization, Data curation, Formal analysis, Funding acquisition, Investigation, Methodology, Visualization, Writing – original draft, Writing – review & editing. LG-R: Conceptualization, Investigation, Methodology, Visualization, Writing – original draft, Writing – review & editing. SC: Visualization, Writing – review & editing. AC-R: Data curation, Resources and Writing – review & editing. JA-F: Data curation, Resources and Writing – review & editing. AC-T: Data curation, Funding acquisition, Conceptualization, Investigation, Writing – original draft, Writing – review & editing.

## Footnotes

^1^ https://github.com/artic-network/artic-ncov2019

^2^ https://www.epicov.org/epi3
