# Supplementary material for: COVID-19 reinfections in Mexico City: implications for public health
Source: Front Public Health. 2024 Feb 14;11:1321283. doi: 10.3389/fpubh.2023.1321283 (PMC10899476; doi:10.3389/fpubh.2023.1321283)
Supplement: Supplementary file 1 [file Data_Sheet_1.docx]

**COVID-19 Reinfections in Mexico City: Implications for public health**

**SUPPLEMENTARY FIGURES**

**
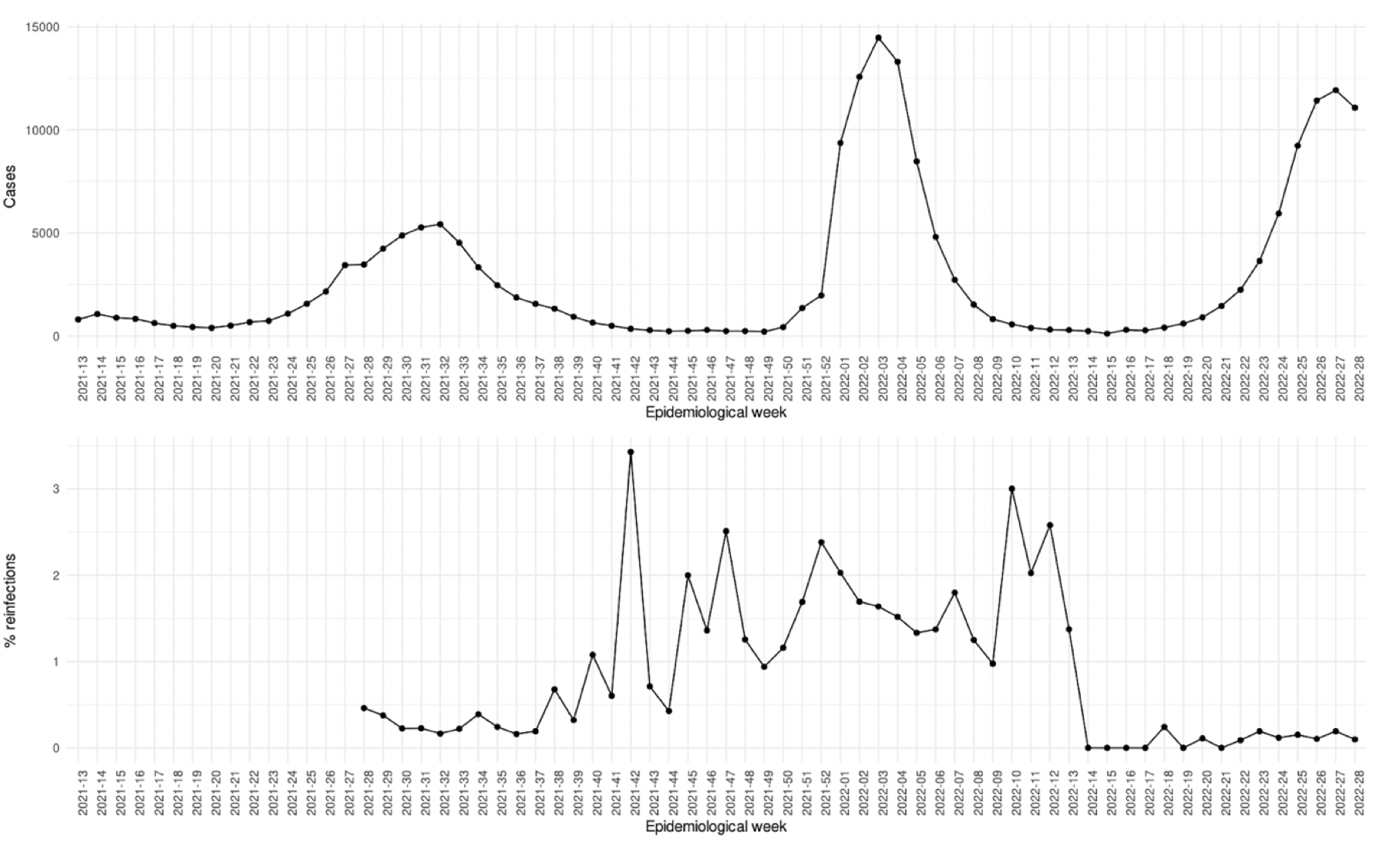
**

Supplementary Figure 1. Number of cases and percentage of reinfections per epidemiological week obtained from SD data.


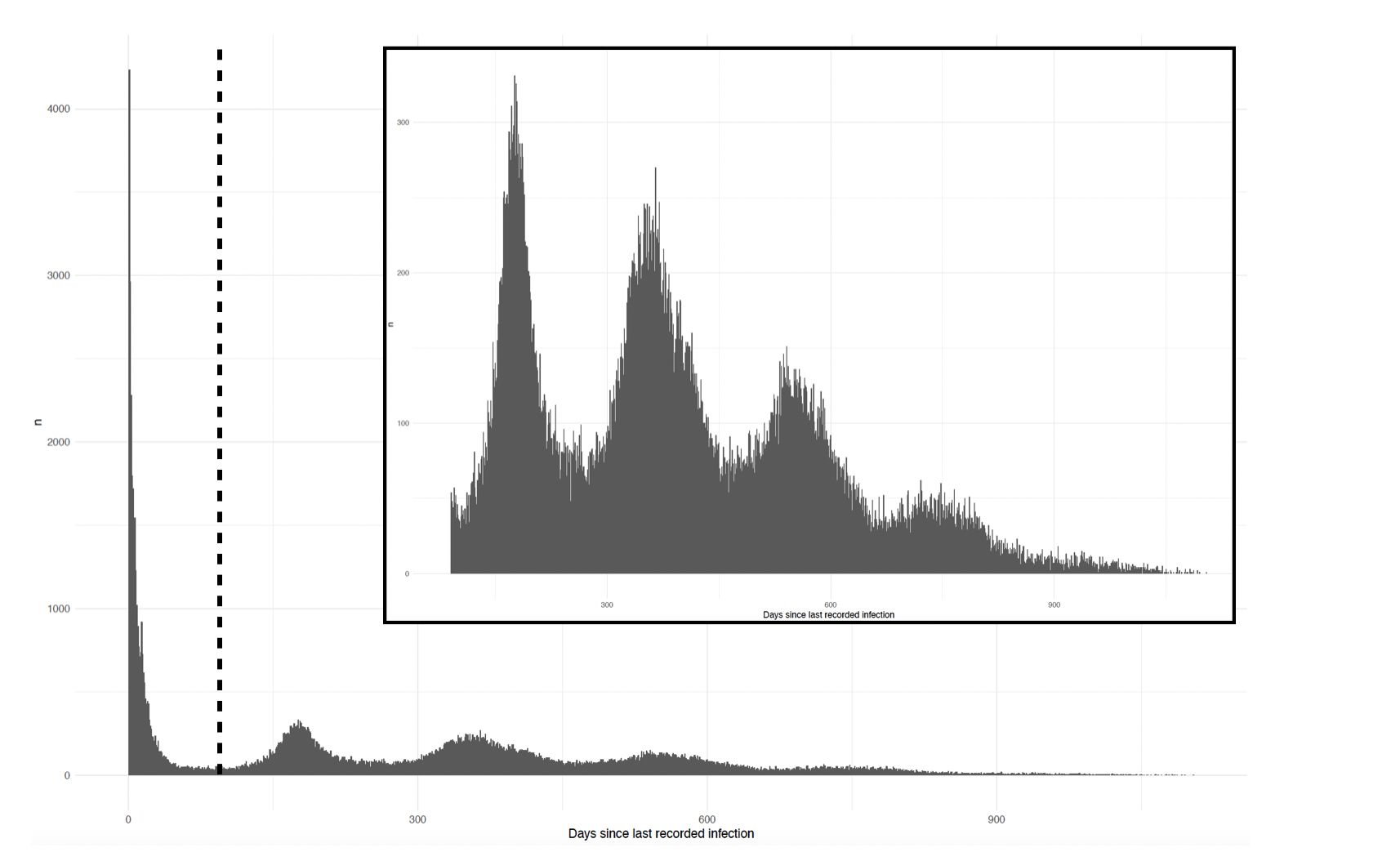


Supplementary Figure 2. The number of reinfections is shown in the y axis and the number of days since last recorded infection (time of separation between infections) is shown in the x axis. The dashed line indicates a time of separation between infections equal to 90 days.


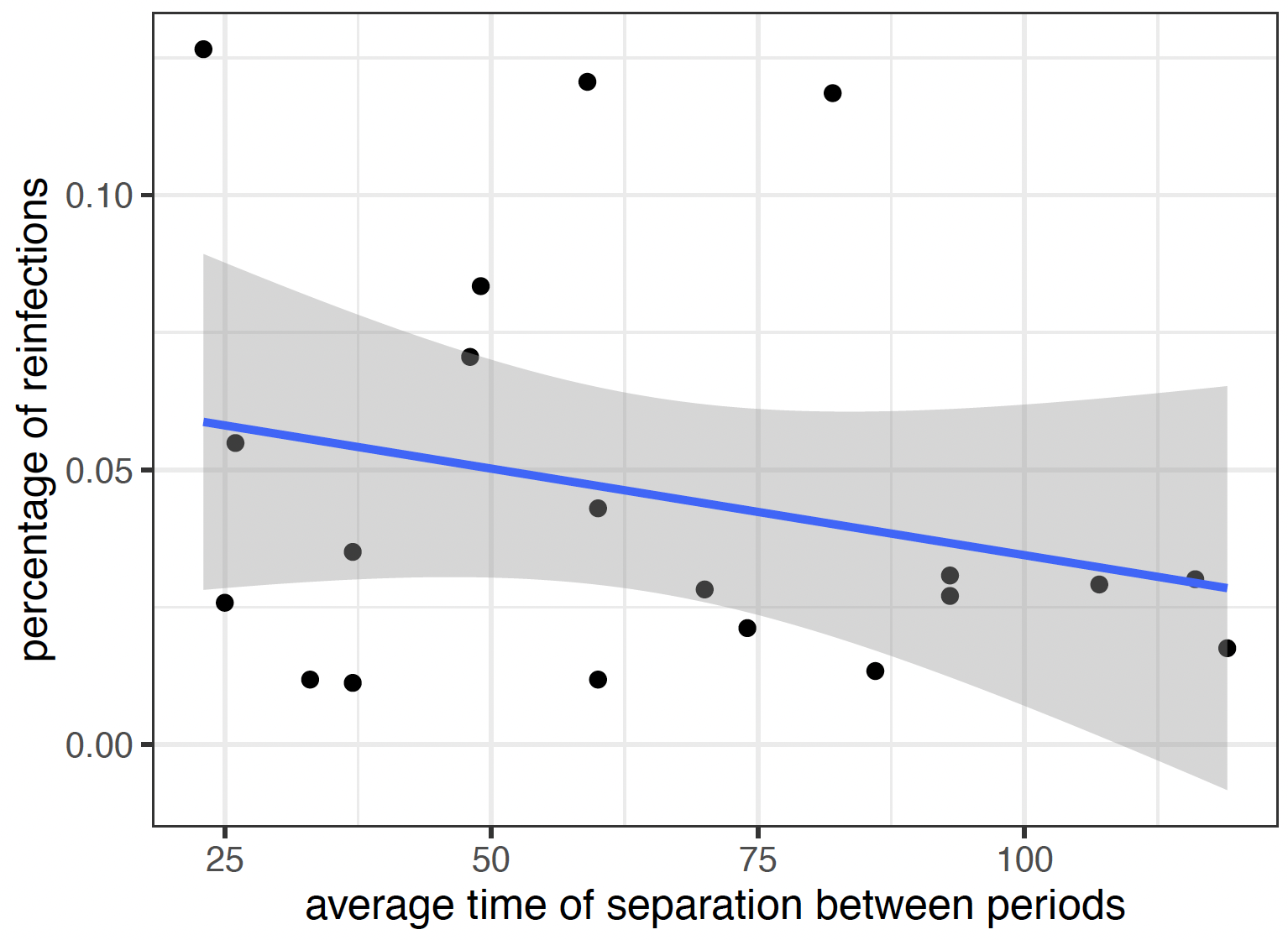


Supplementary Figure 3. Percentage of reinfections observed for each separation time between periods throughout the pandemia. For this analysis, we only considered reinfections occurring on different periods and we only considered pairs of periods with more than 1% of reinfections between them.


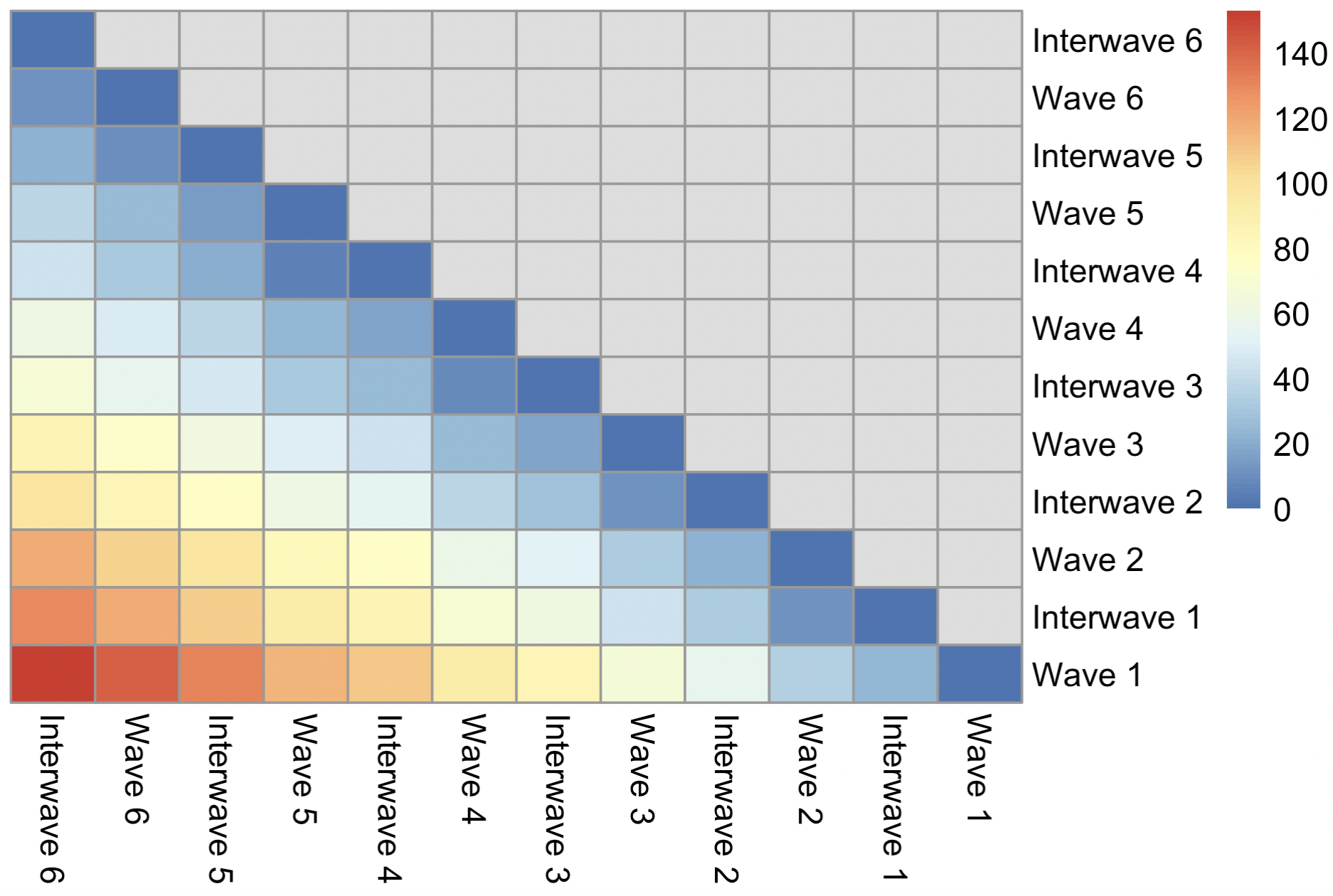


Supplementary Figure 4. Cell X-Y displays the average separation time between waves measured from the start of period X to the end of period Y.


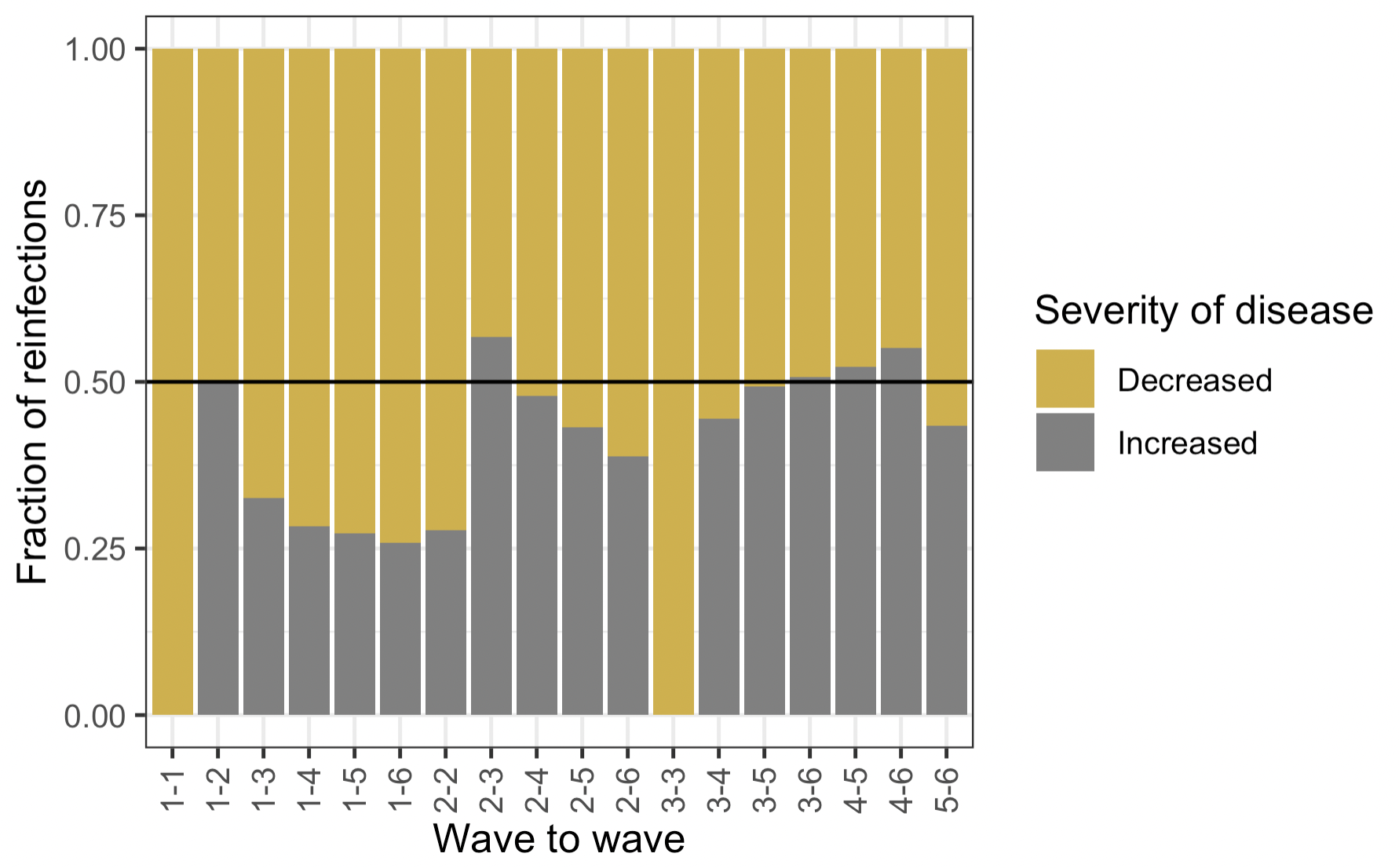


Supplementary Figure 5. Each reinfection was classified as decreased, increased or stable according to the change of severity of the disease from the index infection to the reinfection. The severity of the disease for each infection event was classified as asymptomatic, mild or severe. Only reinfections classified as either decreased or increased are shown in this plot. The bar X-Y displays the cases in which index infection occurred in wave X and reinfection occurred in wave Y.
